# Imaging tip-induced isomerization of naphthalene and azulene moieties

**DOI:** 10.1039/d6cc02979e

**Published:** 2026-07-23

**Authors:** Joel Deyerling, Tzu-Chao Hung, Nicolás Rey, Rémi Pasquier, Leo Gross, Jan Wilhelm, Diego Peña, Jascha Repp

**Affiliations:** a Institute of Experimental and Applied Physics and Halle-Berlin-Regensburg Cluster of Excellence CCE, University of Regensburg 93053 Regensburg Germany joel.deyerling@ur.de jascha.repp@ur.de; b Centro de Investigación en Química Biolóxica e Materiais Moleculares (CiQUS) and Departamento de Química Orgánica, Universidade de Santiago de Compostela 15782 Santiago de Compostela Spain diego.pena@usc.es; c Regensburg Center for Ultrafast Nanoscopy (RUN), Institute for Theoretical Physics, University of Regensburg 93053 Regensburg Germany; d IBM Research 8803 Rüschlikon Switzerland; e Oportunius, Galician Innovation Agency (GAIN) 15782 Santiago de Compostela Spain

## Abstract

Adsorbed in a planar manner on bilayer NaCl(001)/Cu(111), a 1,8-dibrominated naphthalene moiety is debrominated by applying voltage pulses with a scanning tunnelling microscope, and it is subsequently isomerized by additional voltage pulses to a fused 5- and 7-membered-ring system (dehydroazulene moiety). The positions of the 5- and 7-membered rings are demonstrated to be reversibly switchable.

## Abstract 2

This work demonstrates STM-induced isomerization of naphthalene and azulene moieties integrated into an sp^2^-hybridized system adsorbed on an inert substrate.

## Graphical abstract

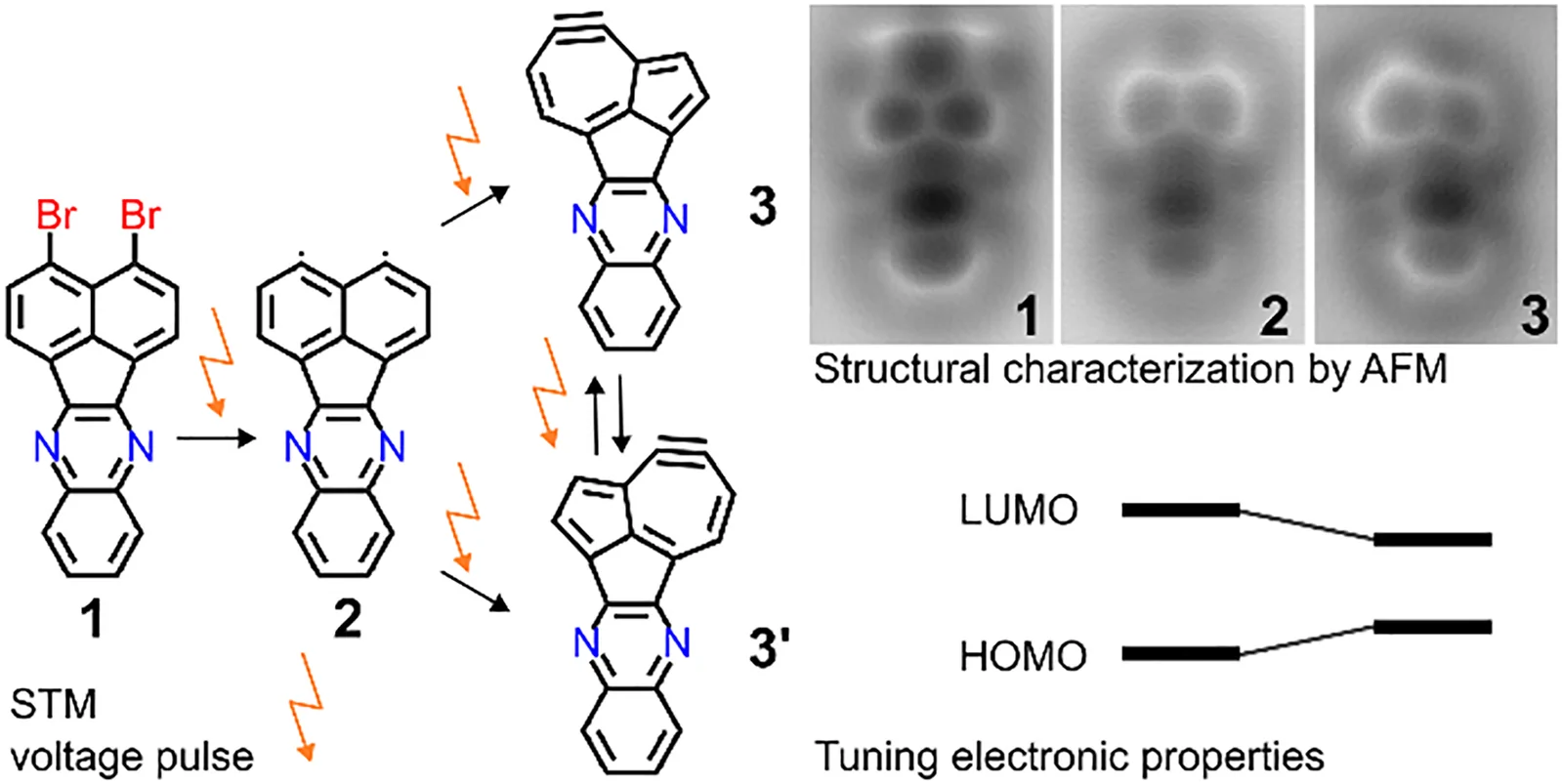

Adsorbed organic structures provide a platform with widely tuneable quantum electronic functionality. Their syntheses on various single-crystalline metallic substrates under ultra-high vacuum (UHV) conditions offer access to compounds^1–7^ that are inaccessible by other means. With this approach, compounds featuring tailored edge states,^8,9^ emergent π-magnetism,^10,11^ controllable transport gaps,^12,13^ and electron correlation^14,15^ have been formed. Azulene moieties enable the embedding of additional functionality related to their non-benzenoid topology: for example, locally increased substrate interaction,^16,17^ an in-plane dipole moment,^16^ and π-magnetism.^18^ Azulene moieties have therefore been utilized in on-surface synthesis: for example, to produce different nanographenes^18–22^ or extended single-layer graphene sheets hosting topological defects.^23^

Azulene has the same number of carbon atoms and bonds as naphthalene, just arranged in a different way, offering fascinating possibilities: Carbon-ring rearrangements between the isomers may be exploited to switch between distinct topologies, reverse dipole moments, tailor transport gaps, electron–electron correlation or – for dehydrogenated compounds – even chemical reactivity. For example, 1,8-dehydronaphthalene shows distinct regioselectivity,^24,25^ while dehydroazulenes may present a novel platform for aryne chemistry.^26,27^

It has been demonstrated that carbon-ring rearrangements can be controllably induced by applying voltage pulses with the tip of a scanning tunnelling microscope (STM) to organic molecules adsorbed on thin-film NaCl.^28–31^ This approach enables the study of the reactant, product and possibly metastable intermediates with atomic precision, providing unprecedented control and insights into the reaction mechanisms. Controlling carbon-ring rearrangements by driving currents through carbon-based electronic devices may enable rich switching functionalities in the future.

A previous STM study reported STM-induced switching of a dehydronaphthalene moiety in an out-of-plane geometry, with the reactive moiety facing the tip (Scheme 1a).^32^ The switching was interpreted as reversible isomerization between dehydroazulenes and a dehydronaphthalene diradical based on density functional theory (DFT) calculations. The molecular carbon backbone in this case was connected by [2.2.2]propellane (sp^3^ carbons), disrupting the π-conjugation. Considering the advantageous properties of sp^2−^ carbon architectures like small bandgaps and efficient charge transport, integration of such a dehydroazulene-based switch in an sp^2−^ carbon system should be rewarding. Furthermore, the delocalized nature of the orbitals may enable switching through charge injection at the inactive part of the molecule. Importantly, it remained to be demonstrated whether the reported dehydronaphthalene/dehydroazulene switch^32^ also operates when the reactive moiety is adsorbed in a planar manner on a substrate.

**Scheme 1:**
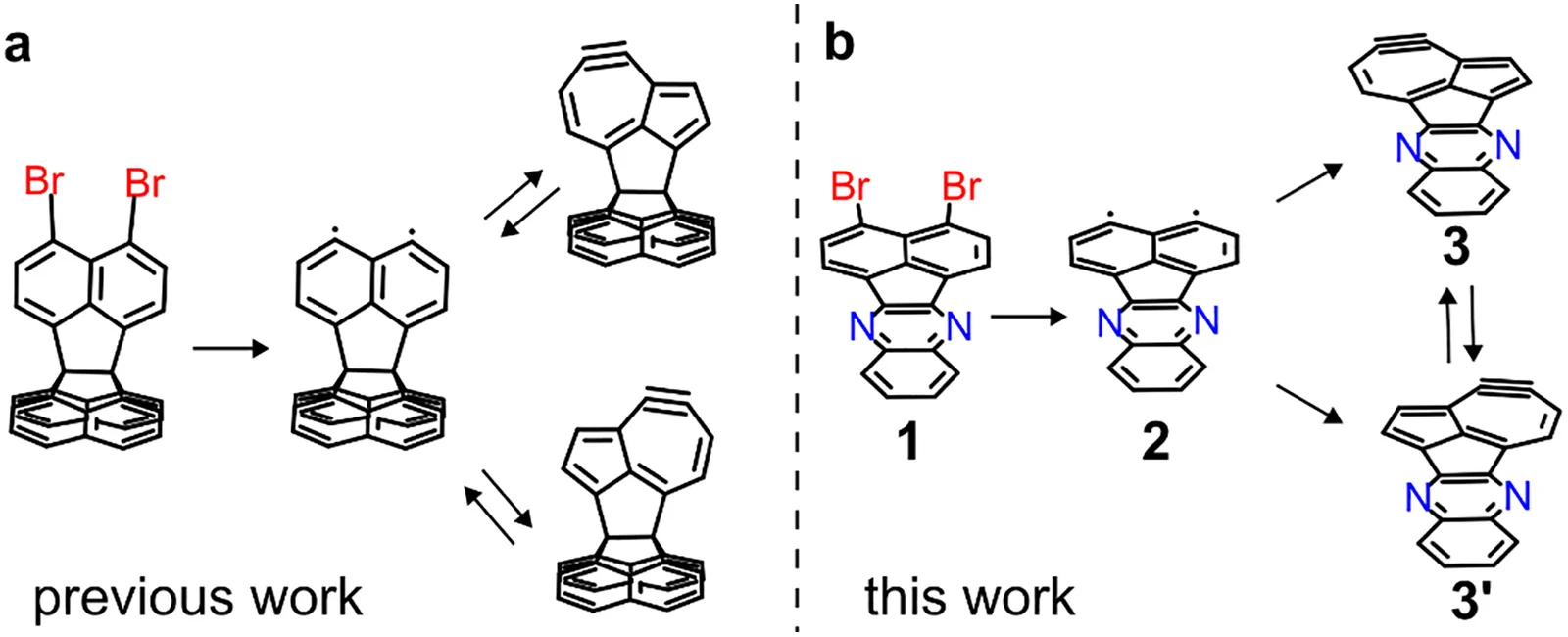
Overview of the tip-induced formation of diradical naphthalene and dehydroazulene moieties. (a) Tip-induced generation of the diradical and isomerization to the dehydroazulene in a molecule where the reactive moiety faces the STM tip.^32^ (b) Tip-induced generation of the diradical and isomerization to the dehydroazulene in a molecule where the reactive moiety is adsorbed in a planar manner on bilayer NaCl(001)/Cu(111). 3 and 3′ are prochiral isomers that are distinguished by the presence of the surface on which they are adsorbed.

For our study, we designed molecule 1 (3,4-dibromoacenaphtho[1,2-*b*]quinoxaline, Scheme 1b) with the same dibrominated naphthalene moiety as in reference.^32^ Importantly, by design, 1 is planar, and all the C and N atoms are sp^2^ hybridized. 1 is expected to be adsorbed flat on the surface, enabling imaging of the carbon rings by bond-resolved non-contact atomic force microscopy (AFM)^33^ as well as characterization of the π-orbitals. Bilayer NaCl(001)/Cu(111) was used as a relatively inert substrate. In the following, we demonstrate the formation of the molecule hosting the diradical naphthalene moiety (2) by tip-induced debromination of 1, the transformation of 2 to dehydroazulene (3/3′) and reversible switching between the pair of adsorption-induced chiral isomers 3 ↔ 3′ (Scheme 1b). All structures and isomers are identified by bond-resolved AFM imaging, supported by imaging the negative (NIR) and positive ion resonances (PIR), and theoretical calculations (gas-phase DFT and GW calculations). The observation of reversible switching between the dehydroazulene isomers is rationalized by gas-phase DFT and calculations of the transition barrier.

Experiments were performed in a custom-built low-temperature STM/AFM operated under ultra-high vacuum and at a temperature of around 7 K. The single-crystalline Cu(111) substrate was cleaned by several cycles of sputtering (Ne^+^ ions, *E*_kin_ = 1 keV) and annealing at 500–550 °C. NaCl(001) bilayers were grown on Cu(111) held at room temperature. 1 was sublimated from a silicon wafer directly onto a Cu(111) sample partially covered by NaCl located in the STM head (*T* < 13 K). CO was dosed onto the cold sample and deliberately picked up from NaCl bilayers for tip functionalization. All AFM data were acquired in constant-height mode at *V* = 0 V, using a qPlus force sensor^34^ with a CO-functionalized tip, unless stated otherwise. The parameters at which the feedback was opened above the bilayer NaCl(001)/Cu(111) are stated in the respective figure captions. Throughout the text, *z*_offset_ denotes the tip–sample distance offset applied after opening the feedback. A positive value indicates an increase in the tip–sample distance. The synthesis of 1 is described in the supplementary information (SI) Section 1. Gas-phase DFT calculations were conducted with ORCA (6.0.0)^35^ and GW with CP2K.^36^ A detailed description of the theoretical calculations is provided in SI Section 3.

Characterization of the different molecular species is discussed first, followed by a report of the controlled isomerization of the debrominated molecules.

After sublimation onto bilayer NaCl(001)/Cu(111), 1 is identified by bond-resolved AFM imaging (Fig. 1a). Debromination of 1 upon sublimation is not observed; see the large-scale AFM images in Fig. S2. Notably, 1 predominantly aligns along the polar high-symmetry directions of the bilayer NaCl(001) substrate. As the two N atoms of 1 are observed to be adsorbed roughly on top of the Na atoms of the top NaCl(001) layer (Fig. S3), we assume that the specific alignment of 1 may be (partially) driven by anchoring of N to an Na cation, akin to the oxygen in PTCDA.^37^ N was deliberately introduced to stabilize the molecule on the surface. Both bromines located at the naphthalene moiety can be cleaved at biases *V* > 2.6 V, either by applying voltage pulses with the tip placed above the molecule or by constant-current STM imaging of 1 (even for *I* < 1 pA). The AFM image of the resulting diradical 2 is displayed in Fig. 1b. Similar to 1, compound 2 is also preferentially aligned along the polar direction of NaCl(001); see Fig. S4. Applying bias voltages of *V* = 3.0–3.4 V with the tip located above 1 or 2 produces isomers with a dehydroazulene moiety (Fig. 1c), which are identified by their 7- and 5-membered rings. We assign them to the adsorption-induced chiral isomers 3 and 3′. Additional AFM characterization of 3 and 3′ at different tip heights (Fig. S5) indicates the location of the formal triple bond, discernible by its slightly brighter (more positive Δ*f*) contrast indicative of a larger bond order compared to the other bonds.^30^ We point out that the apparent shape of the 7-membered rings imaged by AFM varies (for example, see Fig. 2, S6 and S7), which we attribute mainly to CO tilting being dependent on the atomic environment.

**Fig. 1:**
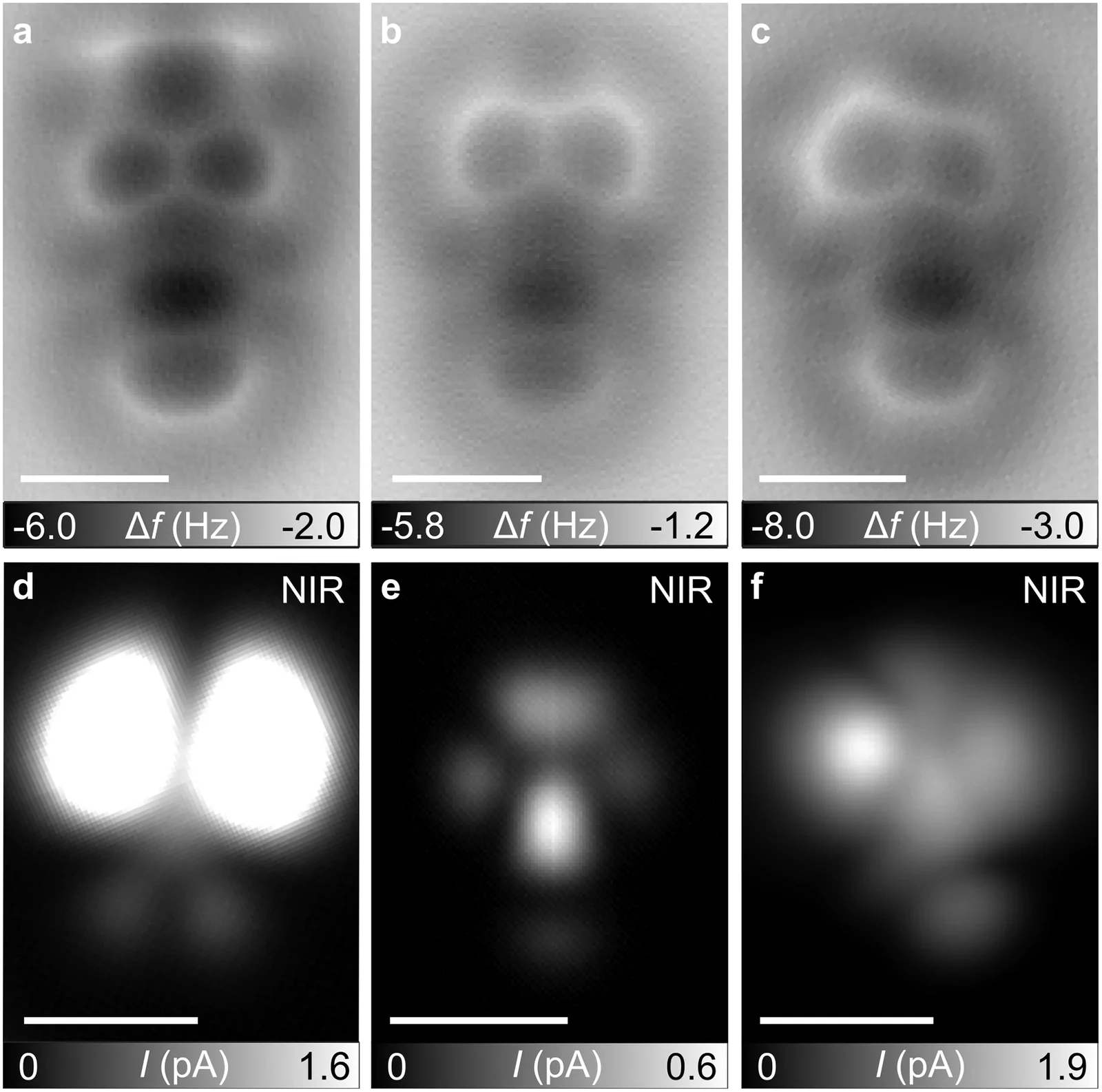
AFM data of 1 (a), 2 (b) and 3 (c) on bilayer NaCl(001)/Cu(111). AFM parameters (a) *V*_set_ = 0.2 V, *I*_set_ = 0.5 pA, *z*_offset_ = 90 pm, (b) *V*_set_ = 0.2 V, *I*_set_ = 1.0 pA, *z*_offset_ = 40 pm, (c) *V*_set_ = 0.2 V, *I*_set_ = 1.0 pA, *z*_offset_ = 0 pm. Scale bars (a)–(c) are 0.5 nm. Constant-height STM data at the NIR (d) of 1 with a metal tip, (e) of 2 with a CO-functionalized tip and (f) of 3 with a CO-functionalized tip. STM parameters (d) *V* = 1.4 V, (e) *V* = 1.6 V, (f) *V* = 1.6 V. Scale bars (d)–(f) are 1.0 nm.

**Fig. 2:**
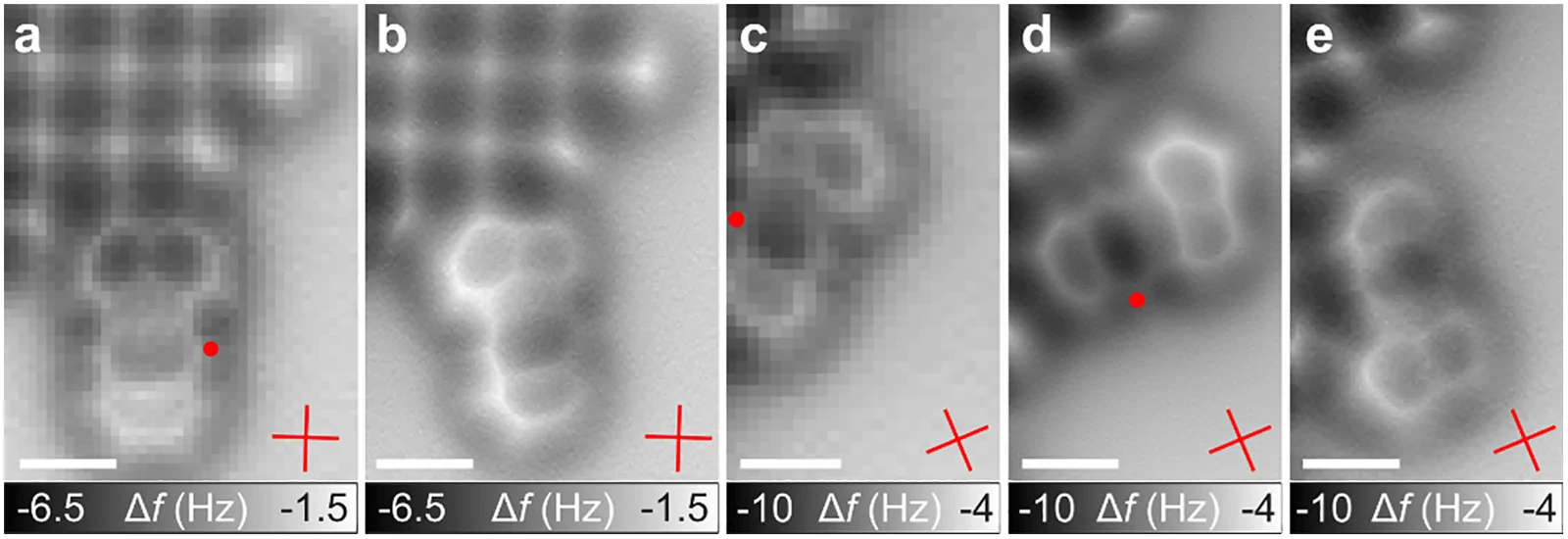
Tip-induced isomerization. (a) AFM image of 2 adsorbed next to the edge of a 3rd-layer NaCl(001) island. (b) AFM image after the voltage pulse showing isomer 3. (c) AFM image of 3′. (d) AFM image of 3 after tip-induced isomerization. (e) AFM image of 3′ after reversed isomerization. The red dots indicate the lateral tip position of the applied voltage pulses. AFM parameters (a) and (b) *V*_set_ = 0.2 V, *I*_set_ = 0.5 pA, z_offset_ = 40 pm, (c) *V*_set_ = 0.2 V, *I*_set_ = 1.0 pA, *z*_offset_ = 35 pm, (d) *V*_set_ = 0.2 V, *I*_set_ = 1.0 pA, *z*_offset_ = 15 pm, (e) *V*_set_ = 0.2 V, *I*_set_ = 1.0 pA, *z*_offset_ = 25 pm. Scale bars are 0.5 nm. The red crosses indicate the polar high-symmetry <110> directions of NaCl(001).

Beyond AFM imaging, each molecular species is also identifiable by the distinct features of the NIR; see the constant-height STM data in Fig. 1d–f. The NIR of 1 (Fig. 1d) is characterized by a node along the long molecular axis, two bright lobes at the dibrominated naphthalene moiety and two fainter lobes at the terminating benzene. The PIR of 1 was not observed (down to *V* = −3.10 V). The NIR of 2 (Fig. 1e), compared to 1, displays a distinctly different nodal structure, albeit with the same *C*_s_ symmetry as 1. The PIR of 2 could not be observed. The NIR of 3 (Fig. 1f) qualitatively bears resemblance to the NIR of 2 with the distinction that the lobe on the 7-membered ring appears more intense, and that the terminating lobe at the benzene is shifted off-centre to the side of the 5-membered ring. Additional data, including the PIR (*V* = −3.0 V) and NIR of 3′ and higher-lying NIRs of 3, are displayed in Fig. S8.

To expand the discussion of the electronic structures of 1, 2 and 3, we conducted gas-phase DFT and GW calculations. We will focus the discussion of the electronic structure of the different compounds on the π-orbitals. For 1, the ground state found in gas-phase DFT calculations is a closed-shell singlet. The gas-phase LUMO orbital (Fig. S9) resembles the experimentally observed NIR. 2 is expected to be in an open-shell configuration. Gas-phase DFT calculations suggest the open-shell singlet as the ground state, but only slightly (38 meV) lower in energy than the triplet. This result is consistent with the previously reported diradical naphthalene moiety attached to a different molecular backbone.^32^ Due to the diradical character of the molecule, it is ambiguous to assign the NIR to a specific orbital calculated by DFT.^38^ Nonetheless, the observed features resemble the SUMOs, LUMO and LUMO+1 of the DFT calculations of 2 (Fig. S9 and Fig. S10). The HOMO–LUMO gap of 2 extracted from DFT calculations is 4.0 eV, and that from GW, accounting for charging, is 6.96 eV. Considering the NIR was observed at approx. 1.6 V, these large values explain why the PIR (HOMO) of 2 could not be observed on NaCl(001)/Cu(111). For 3, gas-phase DFT calculations suggest a closed-shell singlet ground state. The triplet is 1.23 eV higher in energy. The NIR (Fig. 1f and Fig. S8) features are consistent with the calculated DFT LUMO (Fig. S9). Similar agreement can be found for the DFT HOMO and the PIR (Fig. S9 and S8). The optical HOMO–LUMO gap of the closed-shell singlet from DFT is 2.77 eV, and the fundamental gap from GW is 5.55 eV. The experimentally extracted transport gap of 3 on bilayer NaCl(001)/Cu(111) from the onset of orbital contrast as a function of bias in constant-height STM images is approx. 4.5 V. Comparing the HOMO–LUMO gaps of 2 and 3, we conclude that the dehydroazulene compound (3/3′) has a significantly smaller HOMO–LUMO gap, showcasing that the electronic properties can be substantially altered by isomerization switching. The reduction in the HOMO–LUMO gap follows the trend observed for naphthalene *vs.* azulene, related to lower Coulombic repulsion occurring in azulene, due to the reduced spatial overlap of the HOMO and the LUMO.^39^ Note that the HOMO/LUMO of 2 and 3 locally have the same character as those of naphthalene and azulene HOMO/LUMO, respectively. Accordingly, the HOMO–LUMO gap reduction from 2 to 3 can be associated with the same effect.

Turning to controlled tip-induced isomerization, we note that molecules adsorbed next to 3rd-layer NaCl(001) islands (Fig. 2) or next to bromine adatoms (Fig. S6) were investigated for practical reasons: this stabilized the molecules against undesirable translation and rotation.

To induce controlled isomerization, the tip was placed on top of one of the N atoms. Surprisingly, this was the only lateral tip position at which we could induce isomerization with the following manipulation protocol. The feedback was opened at a setpoint of *V* = 0.2 V and *I* = 0.5–1.0 pA. From there, the tip–sample distance was increased by *z*_offset_ = 250 to 400 pm. Afterwards, the bias was ramped up to *V* = 3.2 V for several seconds. An abrupt change in the current indicated a manipulation event. An example of this procedure, transforming 2 into 3, is shown in Fig. 2a and b. The orientation of 3 is not aligned with the polar high-symmetry direction of the substrate, *i.e.* the isomerization induced a change in adsorption position. We attribute this to the reduced symmetry of the dehydroazulene moiety. Isomerization from 3 (or 3′) back to 2 is not observed. Instead, we observe reversible switching between the dehydroazulene isomers. This is exemplified in Fig. 2c–e, where isomerization from 3′ to 3 and back to 3′ is imaged consecutively. Note that flipping the whole molecule upside down would also convert 3 to 3′ and *vice versa*. However, such a process has a large energy barrier and, therefore, seems very unlikely.

To qualitatively rationalize these observations, we calculated the transition barriers from 2 to 3 and 3 to 3′ in the gas phase for neutral and anionic species (assuming that the transition is triggered by transient negative charging due to tunnelling into unoccupied orbitals). The lowest energy pathways found are shown in Fig. S11a and b. The transition barrier from 2 to 3 is 1.60 eV, and that from 2^−^ to 3^−^ is 2.00 eV. The barrier from 3 to 3′ is 1.88 eV and that from 3^−^ to 3′^−^ is 2.29 eV. Notably, the pathways from 3 to 3′ and from 3^−^ to 3′^−^ include 2 and 2^−^ as intermediates. The fact that 3 and the anionic species 3^−^ are the lowest-energy structures by 0.28 eV and 0.29 eV with respect to intermediates 2 and 2^−^ on the reaction pathways rationalizes why we did not observe the (presumably short-lived) intermediate 2 once 3 or 3′ had been formed.

In summary, we demonstrated the tip-induced debromination of 1, creating 2, which contains a diradical dehydronaphthalene moiety. Furthermore, we controllably isomerized dehydronaphthalene diradical 2 adsorbed on NaCl bilayers to dehydroazulene 3, demonstrating dehydronaphthalene/dehydroazulene isomerization when the reactive moiety is supported by an inert substrate. Additionally, we reversibly isomerized between 3 and its enantiomeric counterpart 3′. The isomerization is rationalized by DFT and transition-barrier calculations. Furthermore, we showed that isomerization from 2 to 3 strongly reduces the HOMO–LUMO gap, providing a handle to tune electronic properties. In addition, isomers 2 and 3 represent highly reactive compounds, providing a platform for future studies using tip-induced chemistry.

## Conflicts of interest

There are no conflicts to declare.

## Supplementary Material

CC-062-D6CC02979E-s001

## Data Availability

Data supporting this article are included as part of the supplementary information (SI), which provides details about the synthesis of the molecules, computational methods and additional experimental data. See DOI: https://doi.org/10.1039/d6cc02979e.
