# Supplementary material for: Imaging tip-induced isomerization of naphthalene and azulene moieties
Source: Chem Commun (Camb). 2026 Jul 23. Online ahead of print. doi: 10.1039/d6cc02979e (PMC13394857; doi:10.1039/d6cc02979e)
Supplement: CC-OLF-D6CC02979E-s001 [file CC-OLF-D6CC02979E-s001.pdf]

Supplementary Information (SI) for Chemical Communications.
This journal is © The Royal Society of Chemistry 2026

Supplementary Information

Imaging Tip-Induced Isomerization of Naphthalene and Azulene Moieties

Joel Deyerling,*[a] Tzu-Chao Hung,[a] Nicolás Rey,[b] Rémi Pasquier,[c] Leo Gross,[d] Jan Wilhelm,[c] Diego Peña*[b,e] and Jascha Repp*[a]

[a]Institute of Experimental and Applied Physics and Halle-Berlin-Regensburg Cluster of Excellence CCE, University of Regensburg, 93053 Regensburg (Germany)

[b]Centro de Investigación en Química Biolóxica e Materiais Moleculares (CiQUS) and Departamento de Química Orgánica, Universidade de Santiago de Compostela, 15782 Santiago de Compostela (Spain)

[c]Regensburg Center for Ultrafast Nanoscopy (RUN), Institute for Theoretical Physics, University of Regensburg, 93053 Regensburg (Germany)

[d] IBM Research, 8803 Rüschlikon (Switzerland)

[e]Oportunius, Galician Innovation Agency (GAIN), 15782 Santiago de Compostela (Spain)

*Corresponding authors

emails:

joel.deyerling@ur.de

diego.pena@usc.es

jascha.repp@ur.de

Table of contents

S1 Synthesis of precursor 1 3

S2 Additional experimental data 5

S3 Computational details and results...12

S1 Synthesis of the precursor 1

General synthetic methods

Starting materials were purchased reagent grade from Sigma-Aldrich and BLDpharm and used without further purification. All reactions were carried out under an inert atmosphere of purified argon using oven-dried glassware. Thin-layer chromatography (TLC) was performed on Silica Gel 60 F-254 plates (Merck). Column chromatography was performed on silica gel (40-60 µm). Nuclear magnetic resonance (NMR) spectra were recorded on a Bruker NEO 750 spectrometer. Mass spectra, using the atmospheric pressure chemical ionization (APCI) method, were recorded on a Bruker MicroTOF spectrometer. Mass spectrum and high-resolution mass spectrum are denoted as MS and HRMS, respectively. Compound **1** was synthesized following a previously reported method developed for similar compounds.[1]

Synthesis of the precursor 1

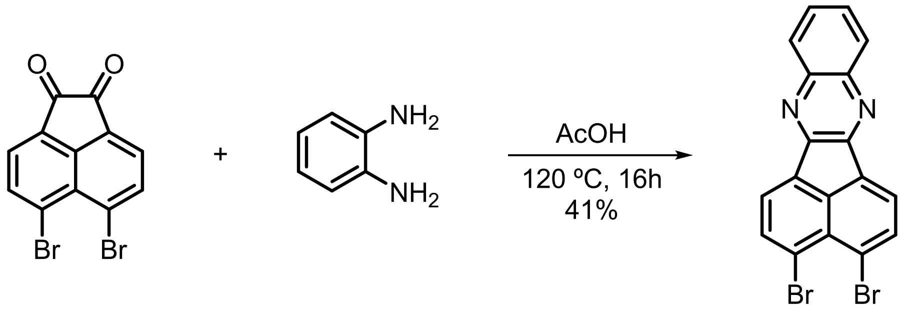

1

A mixture of 5,6-dibromo-1,2-acenaphthylenedione (50 mg, 0.147 mmol) and *o*-phenylenediamine (19.1 mg, 0.176 mmol) in AcOH (10 mL) was heated at 120 ºC for 16 hours. Then, the mixture was filtered and washed with chloroform to yield compound **1** as a yellow powder (25 mg, 41%).

^{1}H NMR (363 K, 750 MHz, $C_2D_2Cl_4$), δ: 8.23 (s, 4H), 8.21 (dd, J = 6.3, 3.5 Hz, 2H), 7.76 (dd, J = 6.3, 3.5 Hz, 2H) ppm.

MS (APCI): m/z: 412 ($[M+1]^+$, 100). **HRMS (APCI)**, m/z found: 412.9131 (calc. for $C_{18}H_9Br_2N_2$: 412.9107).

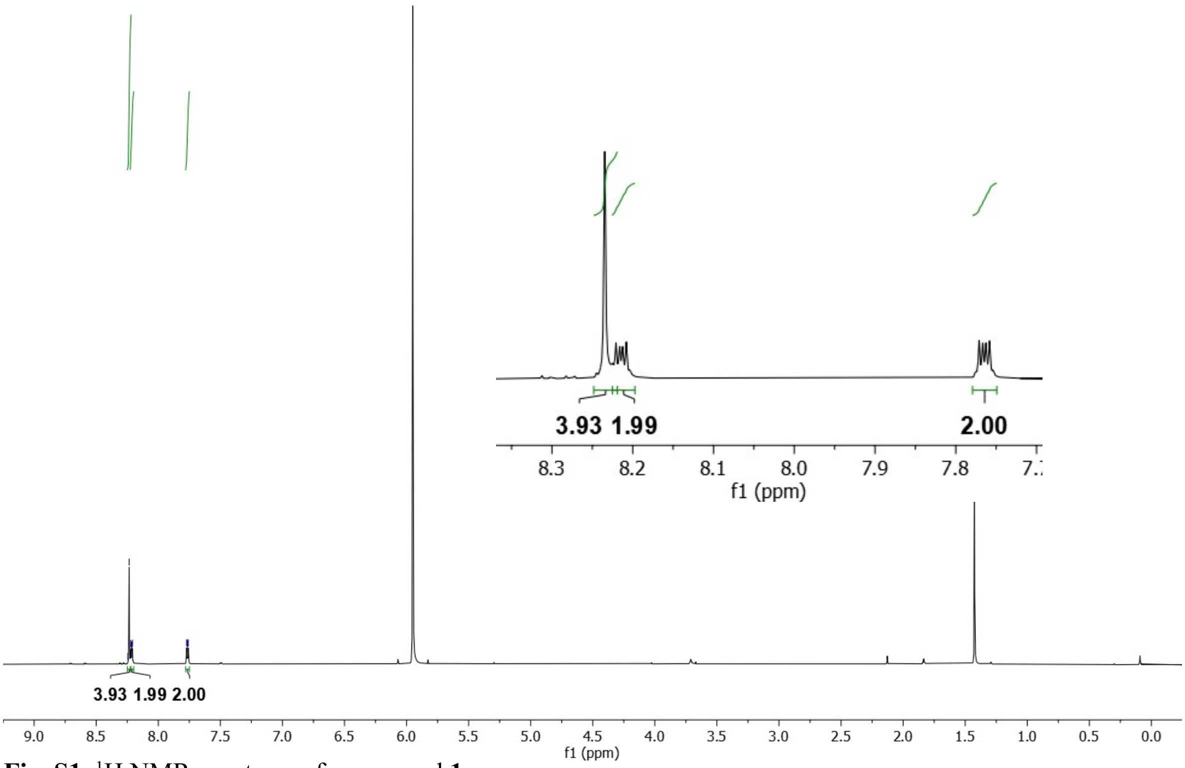

Fig. S1. ^{1}H NMR spectrum of compound **1**.

S2 Additional experimental data

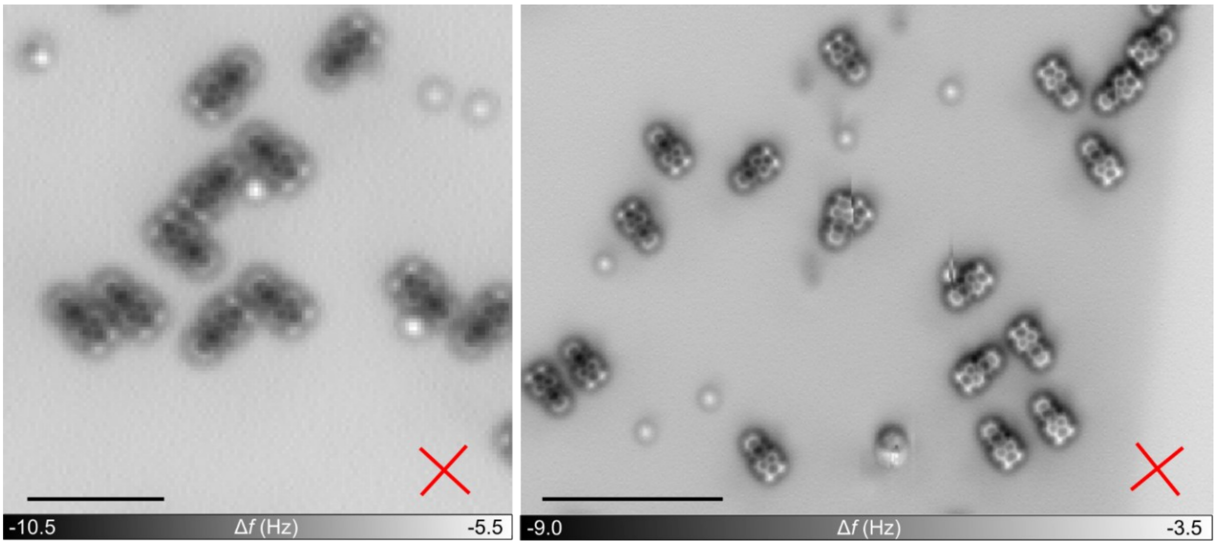

Fig. S2. Large-scale AFM images of **1** adsorbed on bilayer NaCl(001)/Cu(111). AFM parameters V_{set} = 0.2 V, I_{set} = 1.0 pA, z_{offset} = 80 pm (left) and V_{set} = 0.2 V, I_{set} = 0.5 pA, z_{offset} = 60 pm (right). Scale bars are 3 nm (left) and 5 nm (right). The red crosses indicate the polar high-symmetry <110> directions of NaCl(001).

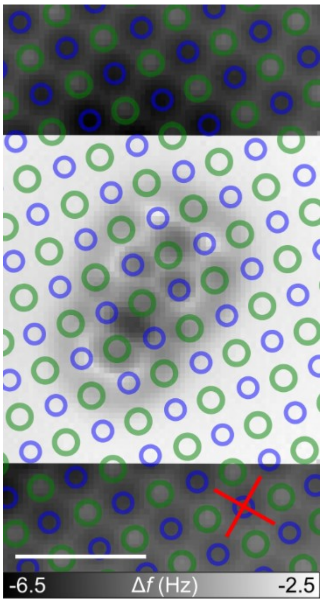

Fig. S3. AFM image displaying the NaCl lattice and **1** to determine the adsorption site. The NaCl(001) lattice is overlaid (green Cl, blue Na). The N atoms of **1** sit on top of Na atoms of the NaCl(001) lattice. The red cross indicates the polar high-symmetry <110> directions of NaCl(001). AFM parameters V_{set} = 0.2 V, I_{set} = 1.0 pA, z_{offset} = -150 pm (top), z_{offset} = 80 pm (middle), z_{offset} = -150 pm (bottom). Scale bar is 1 nm.

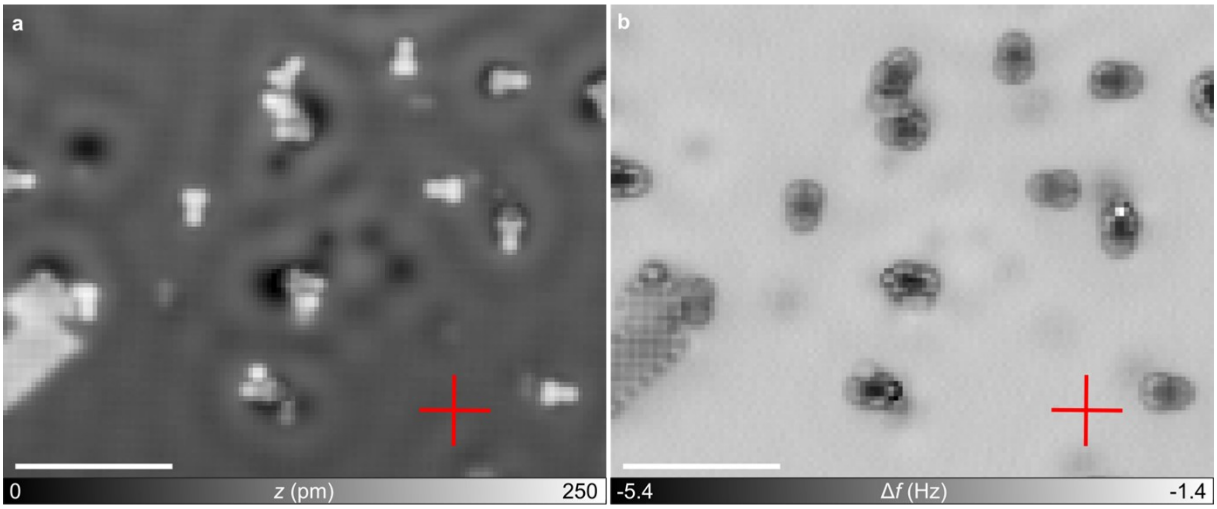

Fig. S4. (a) Overview STM image after debromination of **1** in this area. (b) Corresponding constant-current Δf image. STM parameters V = 0.2 V, I = 0.5 pA (a) and (b). The scale bars are 5 nm. The red crosses indicate the polar high-symmetry <110> directions of NaCl(001).

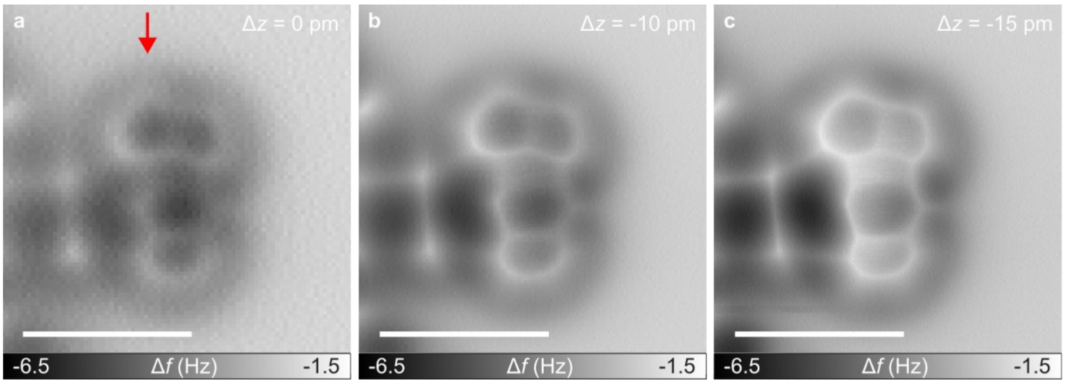

Fig. S5. AFM images of **3** at different tip heights. (a) AFM parameters V_{set} = 0.2 V, I_{set} = 0.5 pA, z_{offset} = 50 pm corresponding to Δz = 0 pm. (b) AFM image at z_{offset} = 40 pm, i.e. Δz = -10 pm. (c) AFM image at z_{offset} = 35 pm, i.e. Δz = -15 pm. All scale bars are 1 nm. The red arrow indicates the position of the formal triple bond.

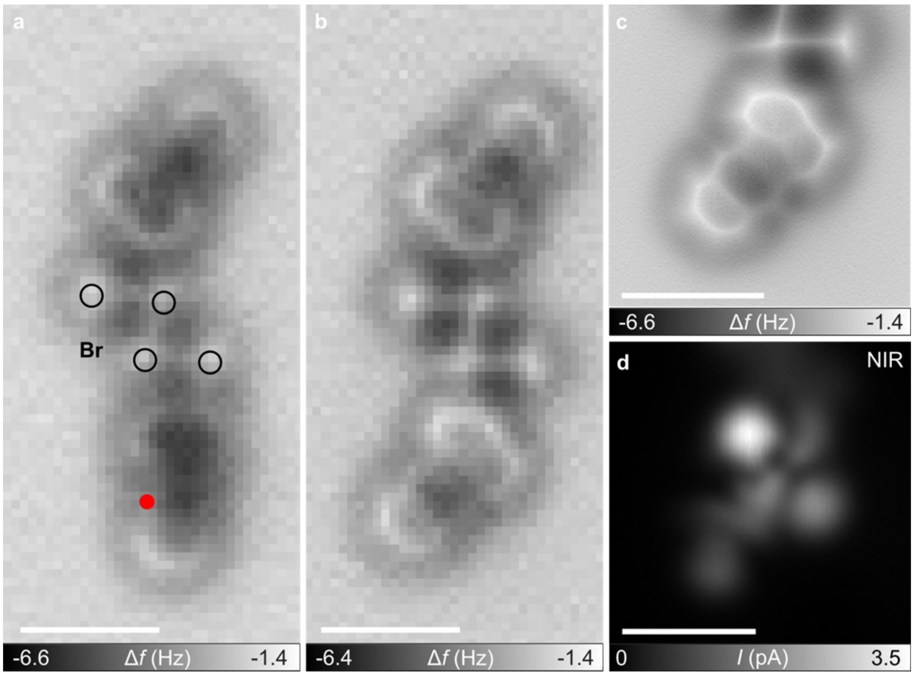

Fig. S6. Tip-induced isomerization from **2** to **3** stabilized next to Br adatoms. (a) AFM image of two **2** molecules adsorbed next to four bromine adatoms. The red dot indicates the lateral tip position during the voltage pulse. (b) AFM image after applying the voltage pulse. (c) High-resolution AFM image of **3**. (d) Constant-height STM image of the NIR of **3**. AFM parameters V_{set} = 0.2 V, I_{set} = 0.5 pA, z_{offset} = 50 pm (a), (b) and (c). STM parameters V = 1.5 V (d). Scale bars are all 1 nm.

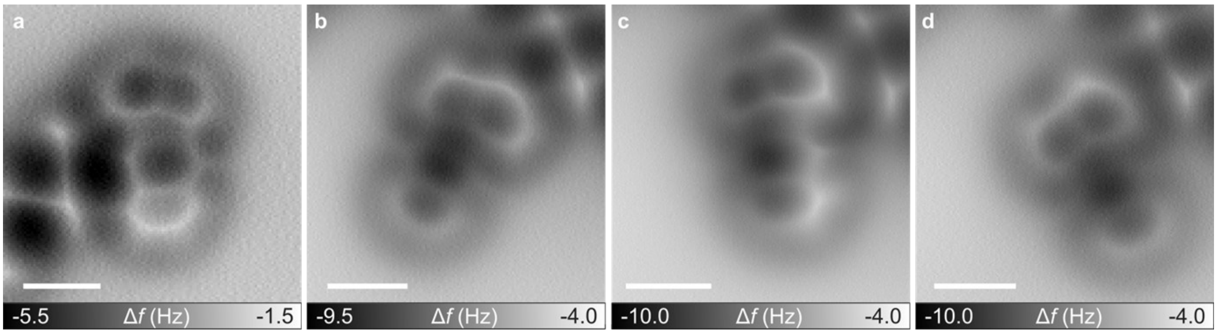

Fig. S7. Overview of AFM images of dehydroazulene isomers displaying different 7-membered ring shapes. (a) AFM image after applying additional voltage pulses to the molecule shown in Figure S6c. (b)-(d) Consecutive AFM images after applying voltage pulses to the molecule. AFM parameters V_{set} = 0.2 V, I_{set} = 0.5 pA, z_{offset} = 0 pm (a), V_{set} = 0.2 V, I_{set} = 1.0 pA, z_{offset} = 60 pm (b) and (c), V_{set} = 0.2 V, I_{set} = 1.0 pA, z_{offset} = 35 pm (d). Scale bars are all 0.5 nm.

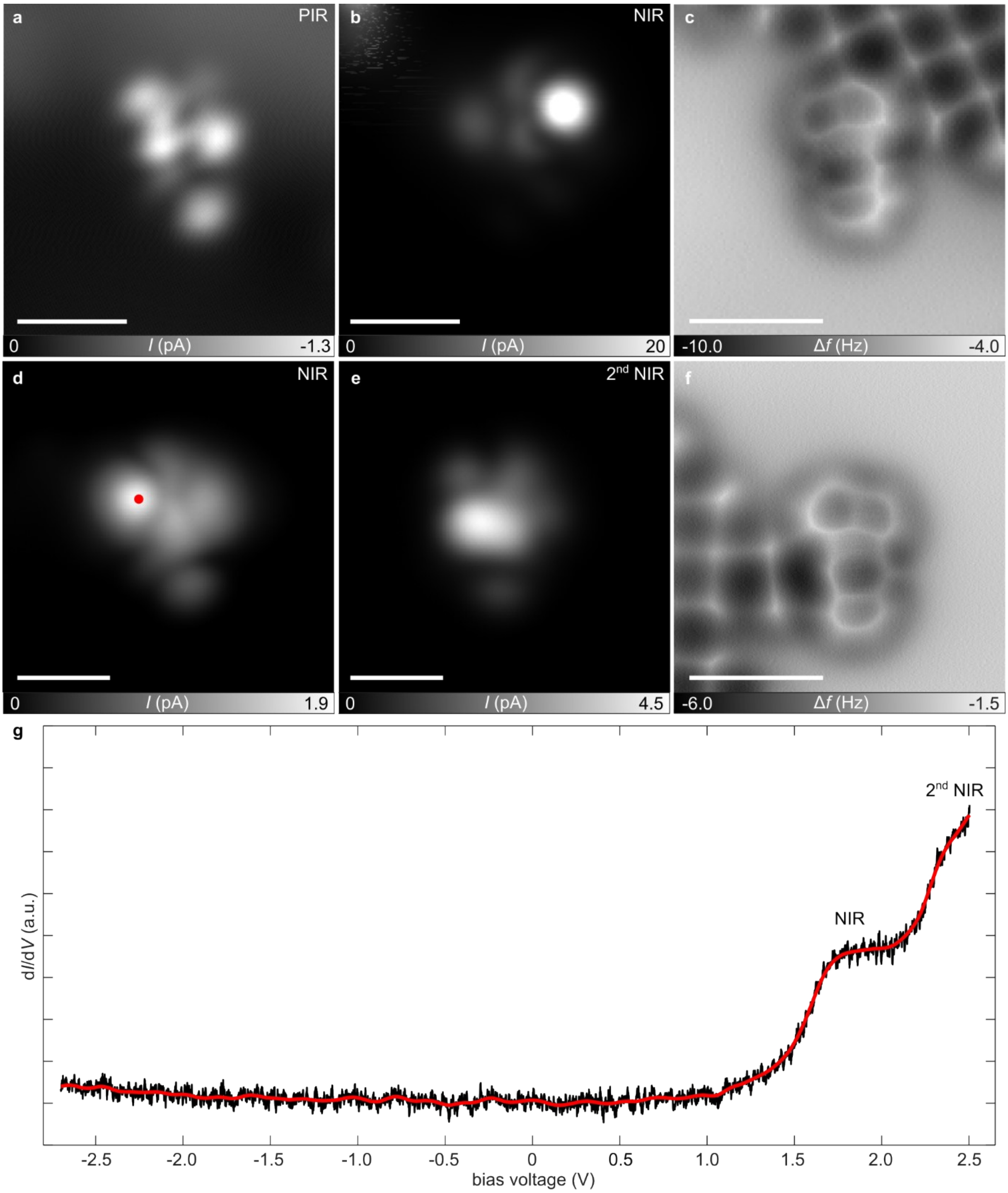

Fig. S8. Additional characterization of the electronic structure of **3** and **3'**. (a) Constant-height STM image of the PIR (V = -3.0 V), (b) constant-height STM image of the NIR (V = 1.65 V) and (c) corresponding AFM image of the dehydroazulene. (d) Constant-height STM image of the NIR (V = 1.6 V), (e) constant-height STM image of the 2nd NIR (V = 2.5 V), (f) corresponding AFM image of the dehydroazulene and (g) dI/dV spectrum on top of the 7-membered ring, see the red dot in (d). STM parameters V = -3.0 V (a), V = 1.65 V (b), V = 1.6 V (d), V = 2.5 V (e). AFM parameters V_{set} = 0.2 V, I_{set} = 1.0 pA, z_{offset} = 25 pm (c) and V_{set} = 0.2 V, I_{set} = 0.5 pA, z_{offset} = 40 pm (f). Scale bars are all 1 nm.

S3 Computational details and results

Gas-Phase DFT calculations were performed with ORCA (6.0.0)[2]. For the geometry optimization and single-point energy calculations the B3LYP functional with def2-TZVP basis set (neutral molecules) and def2-TZVPD (anions) in combination with the def2/J auxiliary basis set and D3 dispersion correction was used. SCF convergence was set to 'VeryTight'. We performed restricted Kohn-Sham DFT calculations and unrestricted Kohn-Sham DFT calculations. For **2** and **3** RIJCOSX was used.

For the transition barrier calculations, the B3LYP functional with def2-SVP basis set (neutral molecules) and def2-SVPD (anions) in combination with the def2/J auxiliary basis set and D3 dispersion correction was used. Transition-state search was conducted using NEB-TS as implemented in ORCA. The values reported in the discussion of the transition barriers in the main text correspond to the values obtained with the def2-SVP and def2-SVPD basis sets. Note, that the difference in Gibbs free energy between **3** and **2** with def2-TZVP is 0.37 eV and between **3**$^-$ and **2**$^-$ with def2-TZVPD is 0.32 eV. The transition barrier heights were also obtained with the def2-TZVP (neutral) and def2-TZVPD (anions) basis sets through transition state geometry optimization. As a starting point the geometries from the def2-SVP (neutral) and def2-SVPD (anions) calculations were used. The transition barrier from **2** to **3** is 1.39 eV (def2-TZVP) and from **2**$^-$ to **3**$^-$ (def2-TZVPD) is 1.83 eV, respectively. From **3** to **3'** (def2-TZVP) it is 1.77 eV and from **3**$^-$ to **3'**$^-$ (def2-TZVPD) it is 2.16 eV.

The GW calculations were performed with CP2K[3,4] using the B3LYP functional in combination with the def2-TZVP all-electron basis set and the corresponding def2-TZVP-RIFIT auxiliary basis set, as provided by the Basis Set Exchange[5]. The Quickstep (GAPW) method was employed with a plane-wave cutoff of 1000 Ry and a self-consistent field (SCF) convergence threshold of 10^{-6}. All calculations were conducted in a $30 \times 30 \times 30$ Å^3 isolated simulation cell. Eigenvalue self-consistent GW_0 ($evGW_0$)[6] calculations were performed. Input and output files for the GW calculations are available in the GitHub[7] and Zenodo[8] repositories.

Table S1. Relative Gibbs free energies of the different DFT calculated configurations of isomers **2** and **3**. The overall lowest energy of all structures is set to dE = 0 eV (**3** closed-shell singlet). The restricted and unrestricted DFT calculations for the singlets of **3** yield the same energies and the same molecular orbitals in a closed-shell configuration.

	**restricted**	**unrestricted**	**unrestricted**
**2**	**1.18 eV**<br><br>**(closed-shell singlet)**	**0.37 eV**<br><br>**(open-shell singlet)**	**0.41 eV**<br><br>**(triplet)**
**3**	**0.0 eV**<br><br>**(closed-shell singlet)**	**0.0 eV**<br><br>**(closed-shell singlet)**	**1.22 eV**<br><br>**(triplet)**

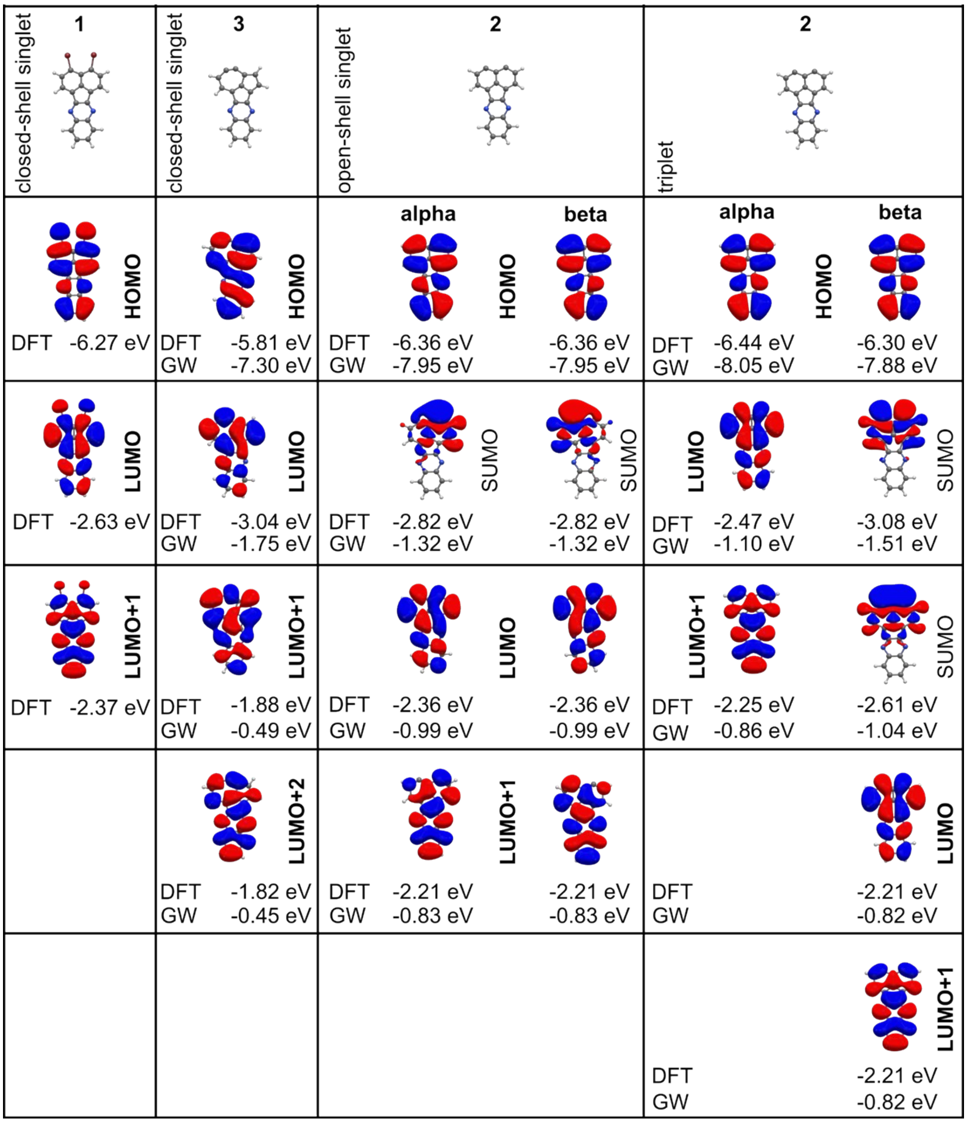

Fig. S9. Overview of the gas-phase DFT calculated orbitals of structures **1** (closed-shell singlet), **3** (closed-shell singlet) and **2** (open-shell singlet and triplet). The energies of the molecular orbitals obtained from the DFT and GW calculations are indicated. For the unrestricted DFT/GW calculations of **2** the alpha and beta orbitals are displayed.

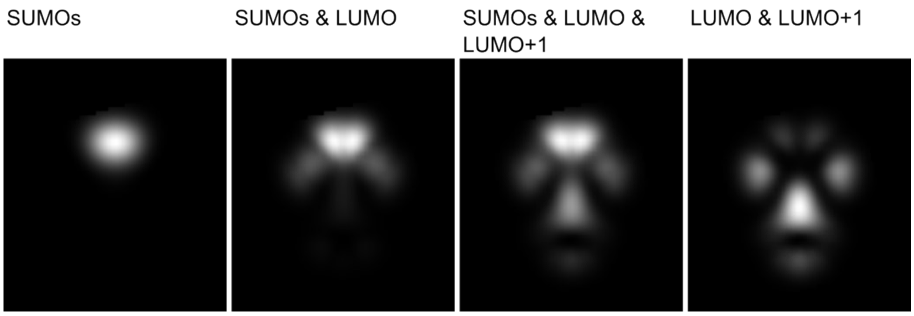

Fig. S10. Constant-height cuts of the isosurfaces of the incoherent sum of DFT-calculated unoccupied orbital densities of **2** (open-shell singlet). The orbital densities were convolved with a gaussian.

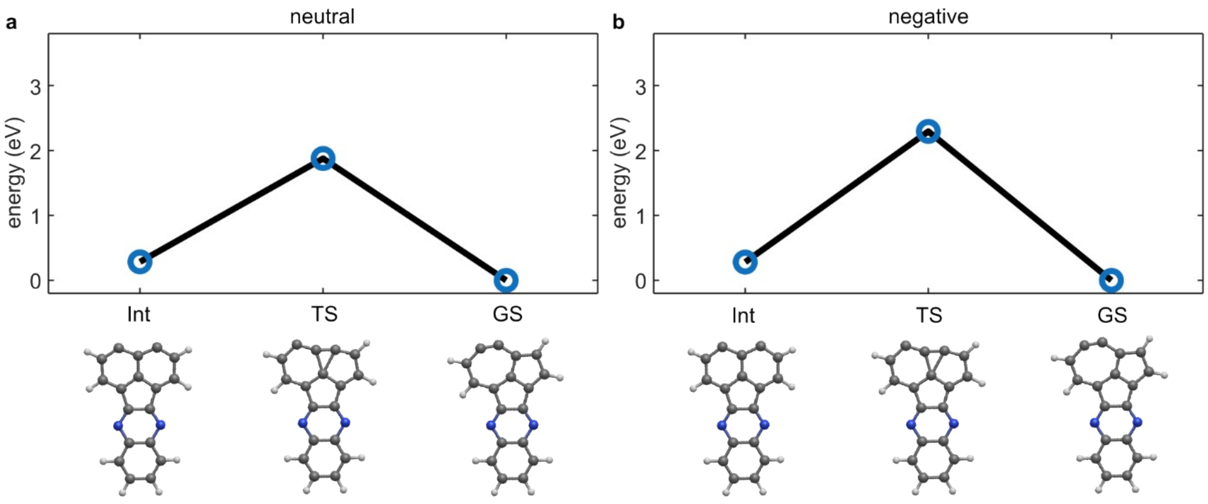

Fig. S11. DFT calculated transition barriers for the isomerization from **2** to **3** in the neutral (def2-SVP basis set) and anionic state (def2-SVPD basis set). (a) Neutral state. (b) Anionic state. GS is the ground state corresponding to **3**, TS the transition state and Int the intermediate state corresponding to **2**.

References

1 E. Mahjoob, A. Khalaj, S. N. Ostad, E. Azizi, S. Fouladdel, S. Tavajohi, R. Salehi and R. Dowlatabadi, Investigation of selective cytotoxicity and determination of ligand induced apoptosis of a new acenaphtho 1,2-b quinoxaline derivative, *Arzneimittel-Forschung*, 2009, **59**, 526–531.

2 F. Neese, Software update: The ORCA program system—Version 6.0, *WIREs Computational Molecular Science*, 2025, **15**, e70019.

3 T. D. Kühne, M. Iannuzzi, M. Del Ben, V. V. Rybkin, P. Seewald, F. Stein, T. Laino, R. Z. Khaliullin, O. Schütt, F. Schiffmann, D. Golze, J. Wilhelm, S. Chulkov, M. H. Bani-Hashemian, V. Weber, U. Borštnik, M. Taillefumier, A. S. Jakobovits, A. Lazzaro, H. Pabst, T. Müller, R. Schade, M. Guidon, S. Andermatt, N. Holmberg, G. K. Schenter, A. Hehn, A. Bussy, F. Belleflamme, G. Tabacchi, A. Glöß, M. Lass, I. Bethune, C. J. Mundy, C. Plessl, M. Watkins, J. VandeVondele, M. Krack and J. Hutter, CP2K: An electronic structure and molecular dynamics software package - Quickstep: Efficient and accurate electronic structure calculations, *The Journal of Chemical Physics*, 2020, **152**, 194103.

4 M. Iannuzzi, J. Wilhelm, F. Stein, A. Bussy, H. Elgabarty, D. Golze, A.-S. Hehn, M. Graml, S. Marek, B. S. Gökmen, C. Schran, H. Forbert, R. Z. Khaliullin, A. Kozhevnikov, M. Taillefumier, R. Meli, V. V. Rybkin, M. Brehm, R. Schade, O. Schütt, J. V. Pototschnig, H. Mirhosseini, A. Knüpfer, D. Marx, M. Krack, J. Hutter and T. D. Kühne, The CP2K Program Package Made Simple, *The Journal of Physical Chemistry. B*, 2026, **130**, 1237–1310.

5 B. P. Pritchard, D. Altarawy, B. Didier, T. D. Gibson and T. L. Windus, New Basis Set Exchange: An Open, Up-to-Date Resource for the Molecular Sciences Community, *Journal of Chemical Information and Modeling*, 2019, **59**, 4814–4820.

6 D. Golze, M. Dvorak and P. Rinke, The GW Compendium: A Practical Guide to Theoretical Photoemission Spectroscopy, *Frontiers in Chemistry*, 2019, **7**, 377.

7 https://github.com/RemiPasquier/public_dyson_orbital.git.

8 https://doi.org/10.5281/zenodo.19224742.
